# Treatment for giant cell tumor of the spine metastasizing to the lung: A report of two cases and a literature review

**DOI:** 10.3892/ol.2014.2837

**Published:** 2014-12-30

**Authors:** AIKEREMUJIANG MUHEREMU, ZHEN HUANG, XIAOHUI NIU

**Affiliations:** 1 Department of Orthopedic Oncology Surgery, Beijing Jishuitan Hospital, Beijing 100035, P.R. China; 2 Medical Center, Tsinghua University, Beijing 100084, P.R. China

**Keywords:** giant cell tumor of the bone, spine, pulmonary metastasis

## Abstract

Giant cell tumors of the bone (GCTB) account for 5% of all primary skeletal tumors. Although the tumors are normally benign, recurrence and metastasis of GCTB does occur. The most usual sites of a primary GCTB lesion are the distal femur and proximal tibia, and ~3% of these metastasize to the lung. Primary GCTB lesions in the spine are rare, and there have been few cases reporting the pulmonary metastasis of GCTB in the spine. The present study reports two cases of thoracic and sacral spinal GCTB lesions with pulmonary metastasis. One of the patients was a 45-year-old male who presented to hospital with gradually worsening pain in the left buttock during the last two years and was diagnosed with GCTB of the sacrum. The other patient was a 30-year-old female who complained of persistent back pain for a year and was also diagnosed with GCTB of the sacrum. Arterial embolization was performed prior to surgery and computer navigation was used during the surgery, resulting in the two patients achieving en bloc resection of their respective tumors, with satisfactory rehabilitation to follow.

## Introduction

Giant cell tumors of the bone (GCTB) are intramedullary bone tumors with benign and locally aggressive pathological and clinical characteristics (1,2). GCTBs account for 5% of primary skeletal tumors and 21% of benign bone tumors (3,4). Although categorized as a benign bone tumor, GCTBs have been reported to have recurrence rates of 8–62% (5,6) and metastatic rates of 1.5–7% (7,8). GCTBs may have a higher prevalence in females than males, as certain studies have reported a female-to-male ratio of 1.3–1.5:1.0 (9,10). The usual primary sites for GCTBs include the distal femur, proximal tibia and distal radius (11,12). Diagnostically, X-ray and computed tomography (CT) scans may show eccentric lytic lesions with cortical extension. Pain, swelling and occasional pathological fractures are the usual manifestations of GCTB (13). There have been few studies on the primary lesions of GCTs of the spine, sacrum and small bones (14), and even fewer reporting the metastasis of these lesions to the lung. The present case study reports two cases of thoracic and sacral spinal GCTB lesions with pulmonary metastasis. Written informed consent was obtained from the patients.

## Case report

### Case one

**Figure 1:**
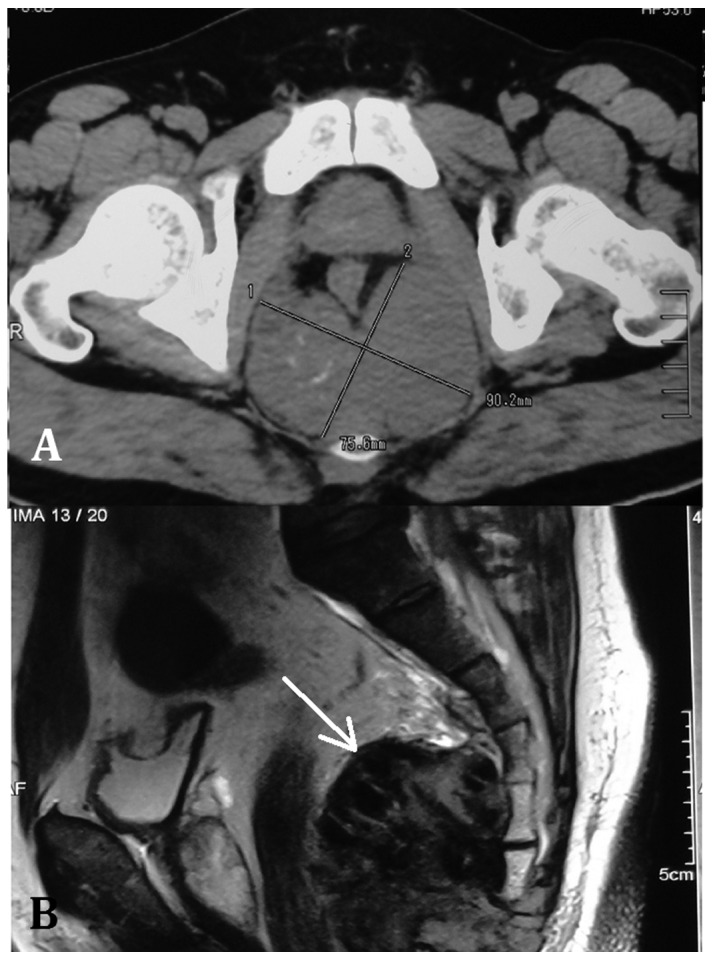
Case one. (A) Transverse and (B) sagittal plane magnetic resonance imaging showing an irregularly shaped mass, 7.5×9.1×9.3 cm in size.

**Figure 2:**
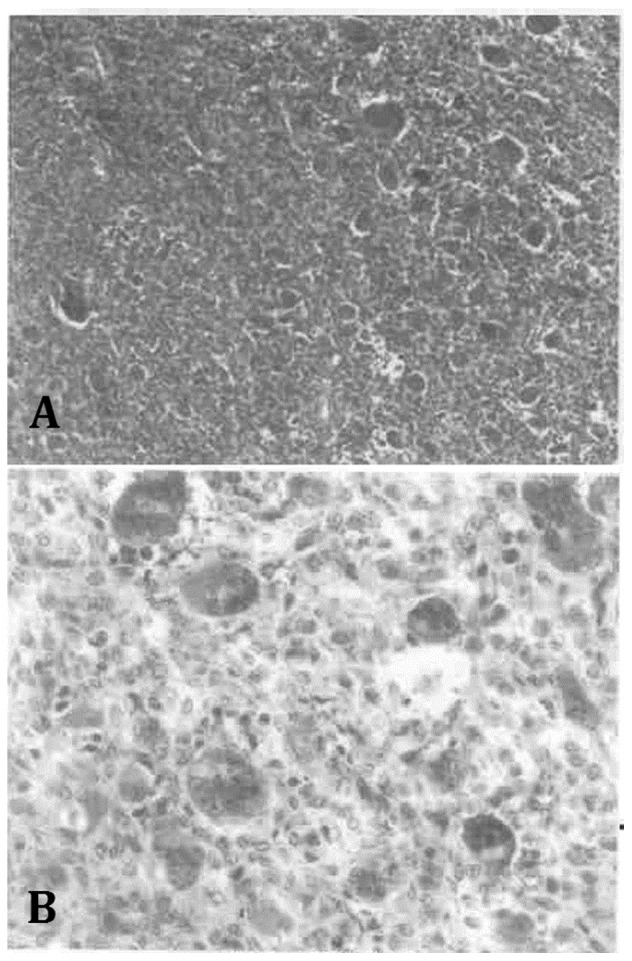
Case one (magnification, ×100). Immunohistochemical staining for the (A) pre-operative fine-needle biopsy and (B) post-operative resection specimens showed the tissues to be AE1/AE3(−), cluster of differentiation 68(+), p53(+) and S-100(+), with a Ki-67 of 20%. The histopathological examinations of the lesion established a diagnosis of a giant cell tumor of the bone.

A 45-year-old male presented to a local hospital with pain in the left buttock that had persisted for 2 years. The pain was particularly bad when the patient was tired, however, the patient did not initially seek medical attention, as the pain was bearable. As the pain gradually worsened over the two-year period, the patient finally attended a clinic at a local hospital. Magnetic resonance imaging showed an irregularly-shaped mass, 7.5×9.1×9.3 cm in size (Fig. 1). Surgeons in the hospital carried out resection of the tumor and sacrum at the level of the 4th sacral vertebra. Immunohistochemical staining for the pre-operative fine-needle biopsy and the post-operative resection showed the lesion to be AE1/AE3(−), cluster of differentiation 68(+), p53(+) and S-100(+), with a Ki-67 of 20%. The histopathological examinations of the lesion established the diagnosis of a GCTB (Fig. 2).

**Figure 3:**
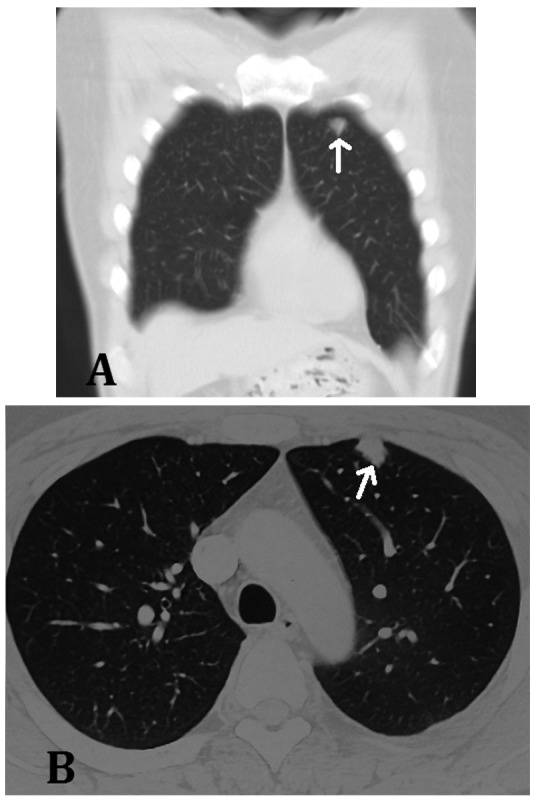
Case one. (A) Coronal and (B) transverse computed tomography scans of the chest, performed four months after the first surgery at the follow-up visit, showing a nodule with clear borders in the anterior upper left lobe of the lung.

**Figure 4:**
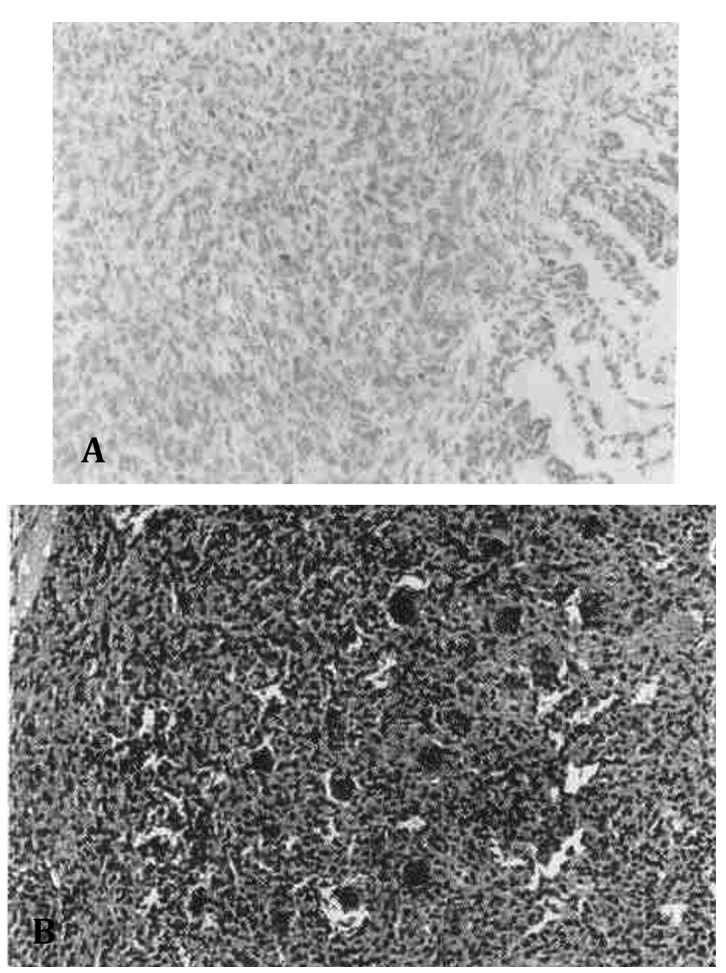
Case one (magnification, ×100). Subsequent to performing a fine-needle biopsy, the pulmonary lobe, which was 7.5×2.5×2.5 cm in size and contained the 2.0×1.0×0.6 cm mass, was resected. Fine-needle and incisional biopsies supported the diagnosis of lung metastases from a giant cell tumor of the bone.

At the follow-up examination four months after the first surgery, a chest CT scan revealed a nodule with clear borders in the anterior upper left lobe of the lung (Fig. 3). Subsequent to a fine-needle biopsy, the 7.5×2.5×2.5-cm pulmonary lobe, which contained the 2.0×1.0×0.6-cm mass, was resected. The fine-needle and incisional biopsies each supported the diagnosis of a GCTB that had metastasized to the lung (Fig. 4).

**Figure 5:**
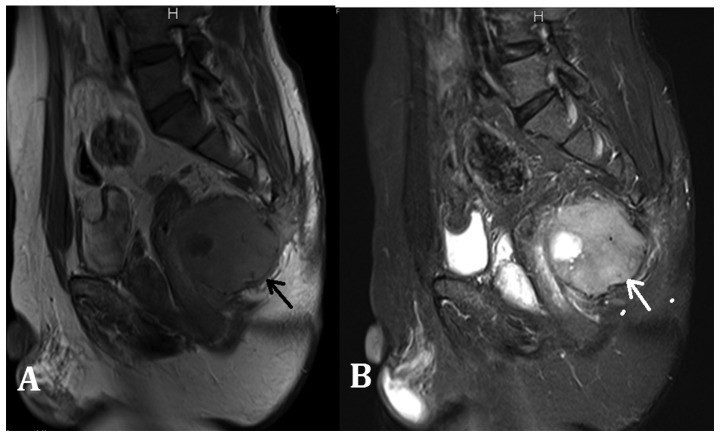
Nine months after the first surgery, a follow-up magnetic resonance imaging scan (A, T1; B, T2) showed a recurrent mass at the site of the original giant cell tumor of the bone.

**Figure 6:**
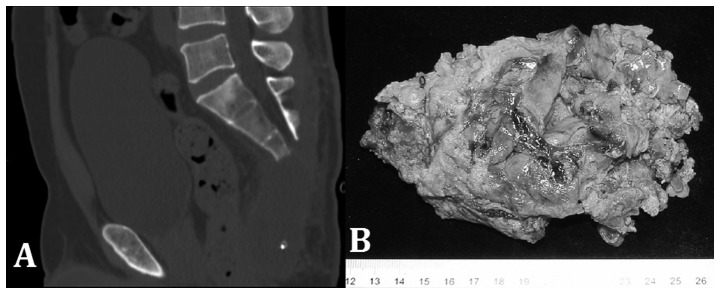
Case one. (A) Postoperative computed tomogaphy scan and (B) gross appearance of the lesion. A surgical resection was performed on the lesion and the sacral body was excised at the level of the 3rd sacral vertebra.

**Figure 7:**
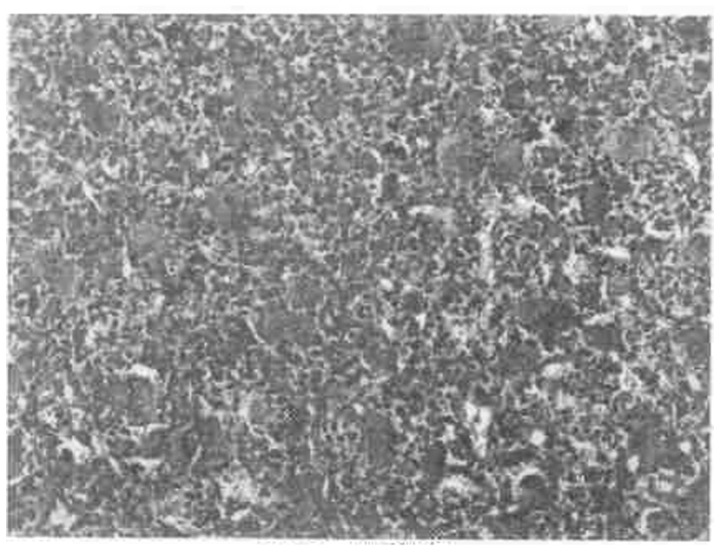
Case one (magnification, ×100). Histopathological analysis performed subsequent to the surgery confirming the presence of a recurrent lesion from the original giant cell tumor of the bone.

Nine months after the first surgery, a follow-up magnetic resonance imaging (MRI) scan showed a recurrent mass at the site of the original GCTB lesion (Fig. 5). The patient was transferred to the Department of Orthopedic Oncology Surgery, Beijing Ji Shui Tan Hospital (Beijing, China) and a surgical resection of the lesion was performed and the sacral body was excised at the level of the 3rd sacral vertebra (Fig. 6). Histopathological analysis after the surgery confirmed that it was a recurrent lesion from the original GCTB (Fig. 7).

**Figure 8:**
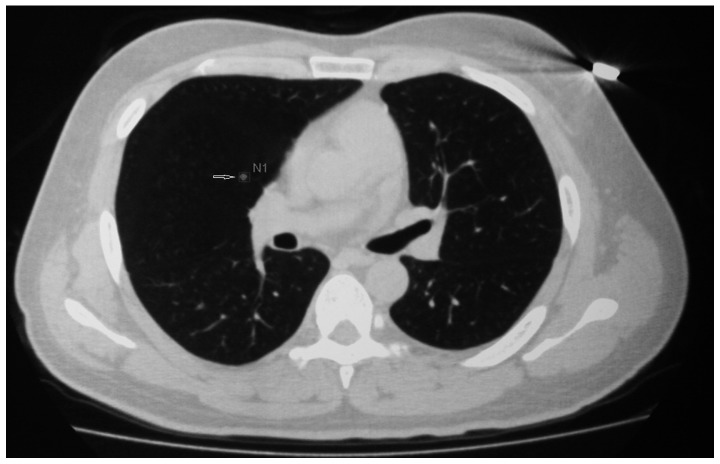
Case one. Chest computed tomography scan performed at a follow-up visit 21 months after the first surgery showing numerous metastatic nodules in diffuse and random distributions in each lung.

**Figure 9:**
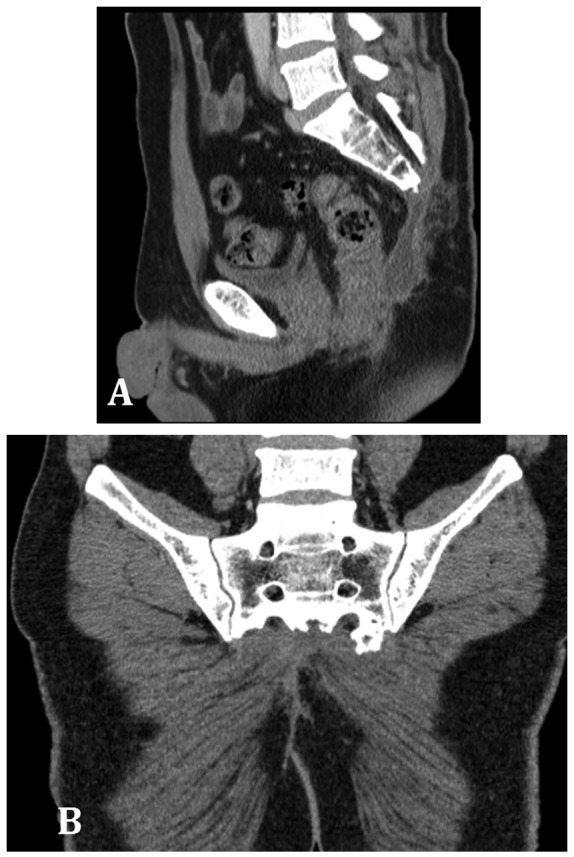
Case one. (A) Sagittal and (B) coronal plane computed tomography scans showing no local recurrence at the last follow-up examination 33 months after the first surgery.

**Figure 10:**
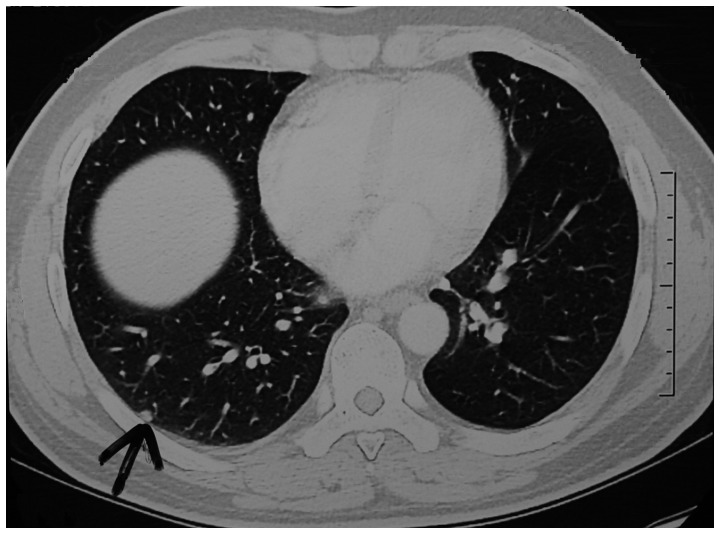
Case one. Chest computed tomography scan showing several newly developed nodules, the largest being 7 mm in diameter, at the last follow-up examination 33 months after the first surgery.

A chest CT scan performed at a follow-up examination 21 months after the first surgery, showed numerous metastatic nodules in diffuse and random distributions in each lung (Fig. 8). The patient refused to undergo any further surgical treatment or chemotherapy. The last follow-up took place 33 months after the first surgery, during which a CT scan found no local recurrence, and the patient complained of no pain at the site of the original lesion (Fig. 9). A chest CT scan showed several newly developed nodules, the largest being 7 mm in diameter (Fig. 10). However, the patient reported no chest pain or trouble in breathing.

### Case two

**Figure 11:**
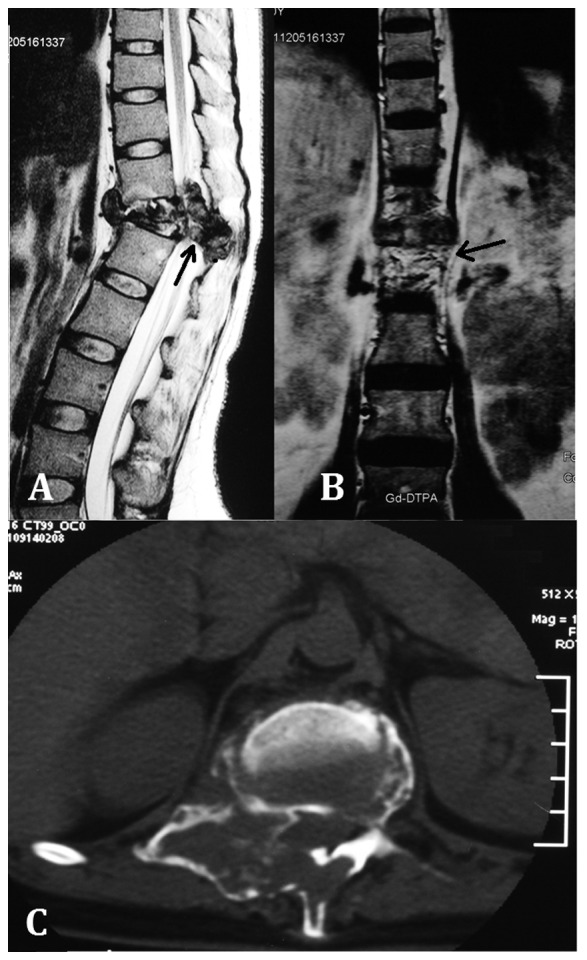
Case two. (A) Sagittal, (B) coronal and (C) transverse plane magnetic resonance imaging showing the additional mass in the spinal canal and posterior column of the T12 vertebra.

**Figure 12:**
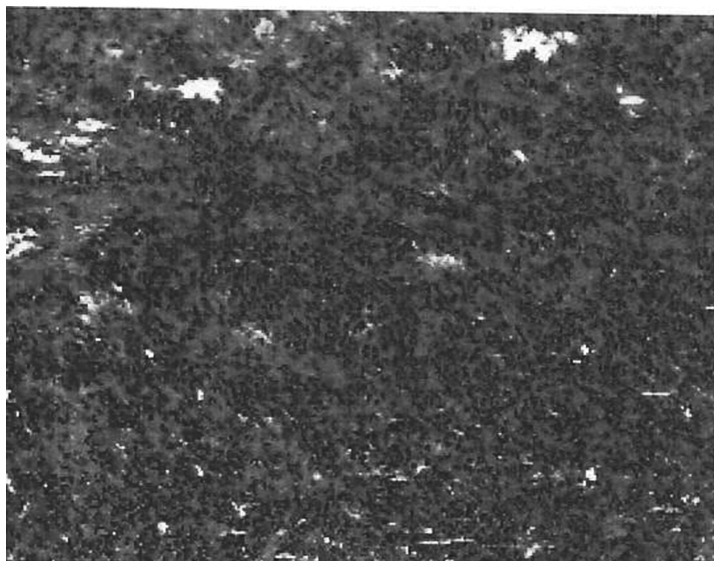
Case two (magnification, ×100). Results of a fine-needle biopsy performed in the local hospital, which resulted in the tumor being diagnosed as a giant cell tumor of the bone.

A 30-year-old female presented to a local hospital due to back pain that had persisted for one year. MRI revealed a 3.2×3.8×3.3-cm lesion in the twelfth thoracic vertebral body, with evident compression of the adjacent spinal canal and foramen. A 3.0×3.4×5.6-cm mass was also present in the spinal canal and posterior column of the T12 vertebra (Fig. 11). A fine-needle biopsy was performed and the tumor was diagnosed as a GCTB (Fig. 12).

**Figure 13:**
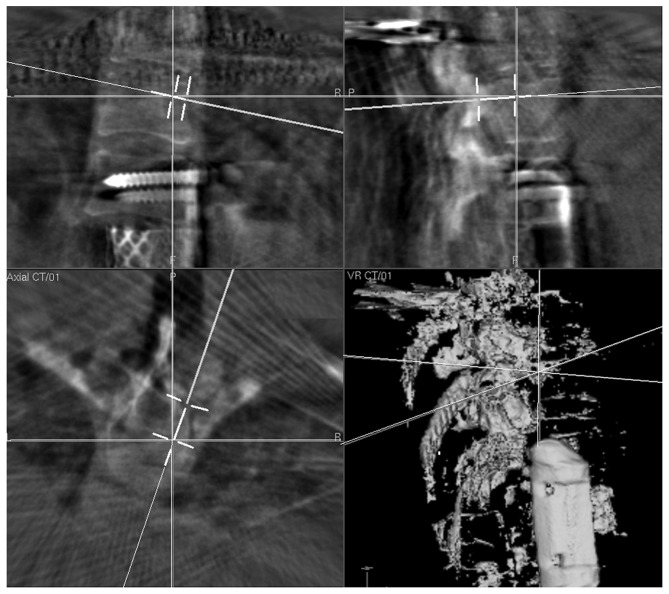
Case two. Images showing the computer-guided resection of the primary tumor.

**Figure 14:**
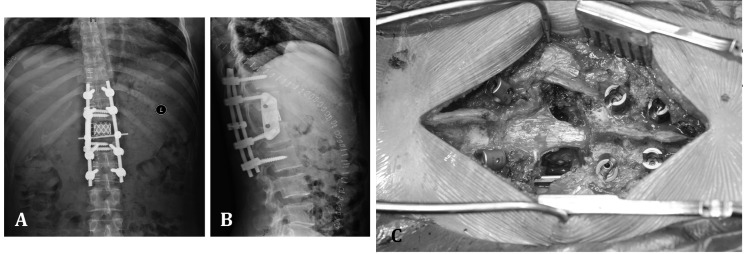
Case two. Postoperative (A) coronal and (B) sagittal computed tomogaphy scans, and (C) gross appearance of the surgical region. The vertebral body of the 12th thoracic vertebra was removed and replaced by a mesh cage filled with bone cement. A titanium palate with four screws was fixed laterally to the 11th thoracic and first lumbar vertebrae to provide reinforcement.

**Figure 15:**
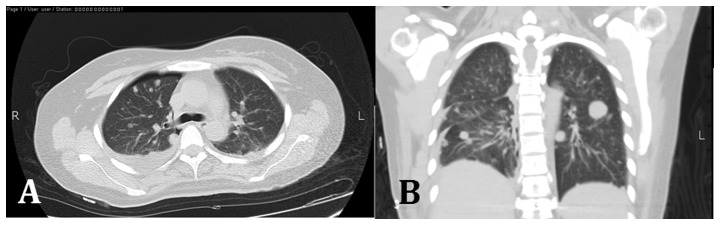
Case two. Post-operative (A) transverse and (B) coronal plane chest computed tomography scans showing multiple nodules of varying sizes in each lung.

The patient was transferred to the Department of Orthopedic Oncology Surgery, Beijing Ji Shui Tan Hospital and computer-guided surgery was subsequently performed to resect the primary tumor (Fig. 13). The spine was stabilized by vertical and horizontal rods fixed by eight pedicle screws fixed into the 10th and 11th thoracic vertebrae, and the 1st and 2nd lumbar vertebrae. The vertebral body of the 12th thoracic vertebra was removed and replaced by a mesh cage filled with bone cement. A titanium palate with four screws was fixed laterally on the 11th thoracic and 1st lumbar vertebrae to provide reinforcement (Fig. 14). Due to the possibility of pulmonary metastasis, a chest CT scan was ordered, which showed multiple nodules of varying sizes in each lung (Fig. 15). Subsequent to a recovery period, the patient was advised to seek further surgical resection in a more specialized hospital or receive resection of the pulmonary metastasis. The patient was subsequently lost to follow-up.

## Discussion

Giant cell tumors of the bone within the vertebrae are rare, accounting for just 2.7–6.5% of all GCTB (15). According to the literature, the sacrum may be the most common site for this lesion, followed by the thoracic, cervical and lumbar segments (16). Patients with vertebral GCTB usually demonstrate clinical manifestations such as pain with radicular distribution, weakness and sensory deficits. A variety of imaging modalities, including magnetic resonance imaging, CT scans, radionuclide imaging and positron emission tomography, are useful tools for the diagnosis for the diagnosis of GCT of the spine. Fine-needle aspiration biopsy can be used to aid the differential diagnosis and confirm the final diagnosis.

The ideal treatment for GCTB consists of an en-bloc excision at the early stages of the development of the lesion (17). However, due to the complicated anatomical structure of the spine and adjuvant spinal tissues, the surgical treatment of tumors of the spine is extremely challenging. In case one of the present study, the local hospital that the patient attended did not have much experience with rare sacral GCTB, and therefore failed to obtain an en-bloc resection. This was probably the most significant cause of the recurrent lesion and lung metastases not long after the first surgical treatment. In our center, the majority of patients are treated by senior surgeons who perform GCTB resection with the assistance of computer navigation, which predominantly achieves en-bloc resection of the tumor. In case one, following the resection of the recurrent lesion in our center, there was no further recurrence at the original site of the tumor and the metastatic lesions in the lungs were relatively stable with no symptoms. In case two, although the patient was lost to follow-up after the first surgery, it is not likely that there will be further recurrence or metastasis.

Although radiotherapy is recommended in cases of unresectable GCTB (18), it is not suitable for vertebral lesions, as it may cause spinal cord myelitis and malignant transformation of the tumor (19,20). Chemotherapy is also not highly recommended for the treatment of GCTB due to its toxic effects and the normally benign nature of GCTB (21). Thus, there is no standard chemical therapy protocol for the lesion. However, denosumab, a novel drug that inhibits the function of the cytokine receptor activator of nuclear factor-κB ligand (RANKL) may be an effective alternative based on the fact that GCTs overexpress RANKL and its receptor (22). Although the two patients in the present study refused to receive chemotherapy, certain other patients with GCTB that metastasized to the lung received a chemotherapy regime consisting of Adriamycin, ifosfamide and mitoxantrone, resulting in more growth of the metastatic tumor in the lung compared with the tumors of those who did not receive chemotherapy (23). However, randomized controlled trials should be carried out to evaluate the pros and cons of chemotherapy for GCTB with pulmonary metastasis.
